# Merging of Azulene and Perylene Diimide for Optical pH Sensors

**DOI:** 10.3390/molecules28186694

**Published:** 2023-09-19

**Authors:** Ping Zhou, Ulrich Aschauer, Silvio Decurtins, Thomas Feurer, Robert Häner, Shi-Xia Liu

**Affiliations:** 1 Department of Chemistry, Biochemistry and Pharmaceutical Sciences, University of Bern, Freiestrasse 3, CH-3012 Bern, Switzerland; zhping@seas.upenn.edu (P.Z.); silvio.decurtins@unibe.ch (S.D.); robert.haener@unibe.ch (R.H.); 2 Department of Chemistry and Physics of Materials, University of Salzburg, Jakob-Haringer-Str. 2A, A-5020 Salzburg, Austria; ulrichjohannes.aschauer@plus.ac.at; 3 Institute of Applied Physics, University of Bern, Sidlerstrasse 5, CH-3012 Bern, Switzerland; thomas.feurer@unibe.ch

**Keywords:** azulene, cyclic voltammetry, computational chemistry, optoelectronic properties, intramolecular charge transfer, perylene diimide, polycyclic aromatic hydrocarbons, ring-fused system, chemical sensors

## Abstract

Polycyclic aromatic hydrocarbons (PAHs) have emerged as promising materials for organic electronics, including organic photovoltaics (OPVs), organic field-effect transistors (OFETs), and organic light-emitting diodes (OLEDs). Particularly, non-hexagonal ring-fused PAHs are highly desirable due to their unique optoelectronic properties. Herein, a new redox-active azulene-perylene diimide triad **1** and its ring-fused counterpart, diazulenocoronene diimide **2**, were synthesized and fully characterized by a combination of NMR, cyclic voltammetry, and UV-visible absorption spectroscopy. Direct comparison of their electronic properties leads us to the conclusion that a significant change in the localization of HOMO and LUMO occurs upon the fusion of azulene and perylene diimide in **2**, leading to the lack of intramolecular charge-transfer character for transitions in the visible spectral region. Density functional theory (DFT) and time-dependent DFT (TD-DFT) calculations were performed to gain further insight into various electronic transitions. Moreover, we found that the adaptive response to acids and bases manifests itself in a reversible two-color change that can be attributed to changes in the chemical structures. Our findings pave the way for manipulating the relative HOMO and LUMO energy levels of organic chromophores by fusing non-alternant azulenes to an otherwise flat PAH, which could possibly lead to applications in organic electronics and optical sensors.

## 1. Introduction

The specific extension of polycyclic aromatic hydrocarbons (PAHs) by embedding non-hexagonal rings to obtain curved π-conjugated structures is an emerging research area but remains a major challenge [1,2,3,4]. Such contorted molecules exhibit enhanced stabilities and intrinsic optoelectronic properties and, thus, are particularly efficient at intermolecular charge transport [5]. Significant effort has been dedicated to the preparation of twisted PAHs in order to figure out the relationship between structure and optoelectronic properties. Our keen interest in the development of organic functional materials in the field of molecular (opto)electronics has led us to take up this challenge. We describe herein a facile synthetic approach that allows the fusion of two azulene rings in a perylene diimide (PDI) chromophore.

Azulene is a non-alternant aromatic π-system consisting of an electron-rich five-membered ring and an electron-deficient seven-membered ring. In stark contrast to its benzenoid isomer naphthalene, azulene shows a large dipole moment, blue color and anti-Kasha fluorescence [6]. It has been widely used for the decoration of rigid π-conjugated skeletons, either linked directly or through a triple bond, to form high-performance semiconducting materials [7,8,9,10,11]. Obviously, the structural extension at the 2- and/or 6-positions of azulene has a significant impact on the stimuli-responsive behavior [12,13,14,15,16] and the optoelectronic performance of the resulting π-functional materials [17]. In general, organic molecules act as hole- and electron-transporting materials when capped with five- and seven-membered rings of azulene, respectively. Despite all these aesthetic achievements, much less is known about azulene-fused PAHs and heteroaromatics prepared from the corresponding azulene precursors [18,19,20,21,22,23,24,25,26,27].

**Scheme 1:**
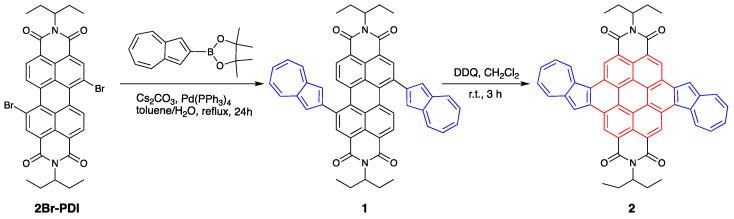
Synthetic routes towards azulene-PDI triad **1** and diazulenocoronene diimide **2** (azulene units are marked in blue and coronene in red).

Among PAHs, PDI has intriguing electronic properties and remarkable thermal, chemical, and photophysical stabilities, leading to a broad range of applications in the field of organic electronics [28,29]. It is also well known as an electron-deficient and light-harvesting component in donor−acceptor ensembles that undergo photo-induced efficient energy and electron transfer reactions [30,31,32,33,34,35,36,37,38,39,40]. The π-expanded PDI with non-alternant aromatic skeletons remains underexplored [17,23,41,42]. As such, in this actual study, we report the rational design and preparation of an azulene end-capped PDI triad **1** and its fused counterpart, diazulenocoronene diimide **2** (Scheme 1). They combine the merits of both PDI and azulene, including intense absorption in the visible spectral region and reversible redox behavior, as evidenced by steady-state absorption measurements and cyclic voltammetry, respectively. The electronic transitions were verified by TD-DFT calculations. Additionally, we demonstrate that they exhibit a reversible colorimetric pH response manifested by an instant color switching between acidic and neutral environments.

## 2. Results and Discussion

### 2.1. Synthesis

As illustrated in Scheme 1, azulene-PDI triad **1** was prepared in 21% yield by the Suzuki–Miyaura coupling reaction of 1,7-dibromo-*N*,*N*′-bis-(1-ethylpropyl)-perylene-3,4,9,10-tetracarboxylic diimide (**2Br-PDI**) [43] with 2-(azulen-2-yl)-4,4,5,5-tetramethyl-1,3,2-dioxaborolane [24] in the presence of Pd(PPh_3_)_4_ as a catalyst and Cs_2_CO_3_ as a base under reflux for 24 h in a toluene–water mixture. The subsequent oxidation of **1** with 2,3-dicyano-5,6-dichloro-1,4-benzoquinone (DDQ) at r.t. in CH_2_Cl_2_ was accomplished to obtain diazulenocoronene diimide **2** in a yield of 40%. Both of them were fully characterized by ^1^H NMR, cyclic voltammetry, UV-Vis-NIR spectroscopy, and high-resolution mass spectrometry. All these analytical data unambiguously correspond to their chemical structures.

### 2.2. Optical Properties

**Figure 1:**
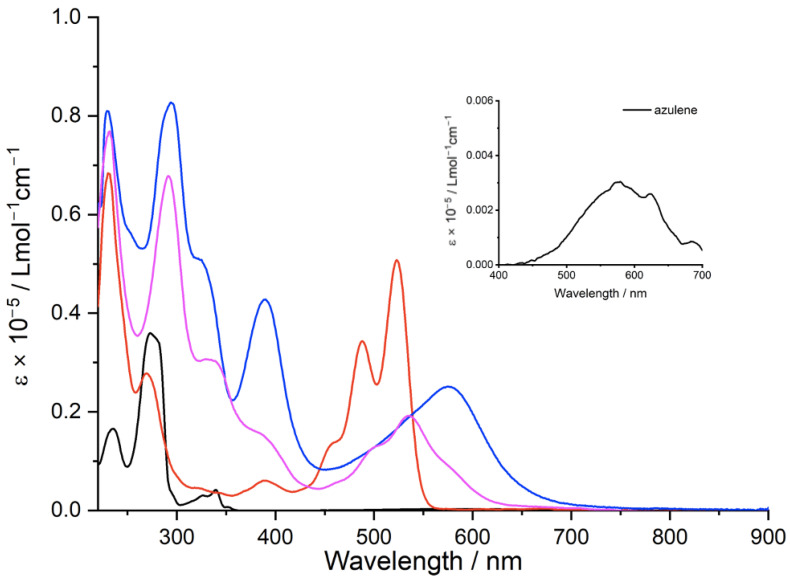
UV-Vis absorption spectra of **1** (blue), **2** (pink), azulene (black), and **2Br-PDI** (red) (*c* = 1 × 10^−5^ M) in CH_2_Cl_2_ at r.t.

As depicted in Figure 1, both **1** (blue curve) and **2** (pink curve) strongly absorb in the UV-Visible spectral region. In comparison with azulene (black curve) and the reference **2Br-PDI** (red curve), intense absorption bands in the whole UV region are assigned to transitions localized on the azulene and PDI components. In contrast to **2Br-PDI**, the triad **1** and the fused counterpart **2** exhibit a series of structureless absorption bands in the spectral range of 400–700 nm with remarkable bathochromic shifts due to electronic interactions between azulene and PDI units enhanced by π-extended conjugation. They are most likely ascribed to a mixture of an intramolecular charge transfer (ICT) from azulene to PDI units and the π–π* transitions localized on the PDI core for **1**, and π-π* transitions delocalized on the diazulenocoronene core for **2**, respectively. Upon fusion, the lowest-energy absorption band centered at 535 nm is hypsochromically shifted by 42 nm. It can be inferred that the resulting diazulenocoronene π-system is distinct from the PDI itself, as rationalized by the theoretical part below. Moreover, the replacement of azulene by its isomeric naphthalene leads to a series of well-structured absorption bands in the visible spectral region [44], particularly a significant hypsochromic shift of the onset of the lowest-energy absorption band. This finding indicates that **2** has a much lower HOMO-LUMO band gap (1.53 eV vs. 2.18 eV) compared to its isomeric analogue dinaphthocoronene diimide, as explained by the following electrochemical properties (Table 1).

**Figure 2:**
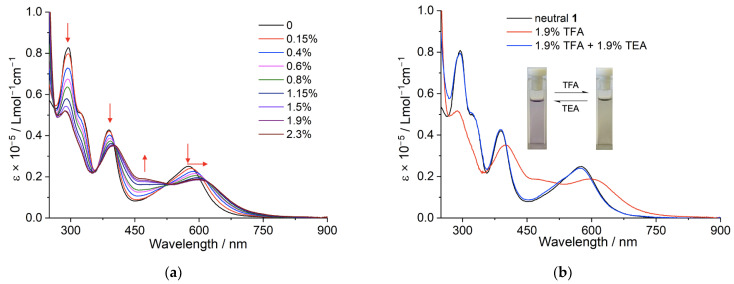
Variation of UV-Vis absorption spectra of **1** (1 × 10^−5^ M) in CH_2_Cl_2_ upon successive addition of TFA (**a**) at r. t. and reversible proton responsiveness (**b**).

**Scheme 2:**
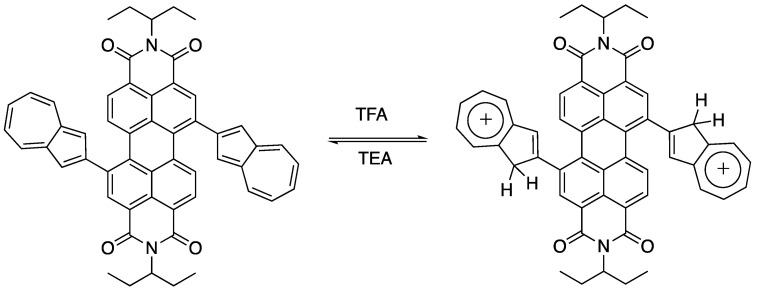
Formation of an azulenium dication of triad **1** upon TFA protonation.

**Figure 3:**
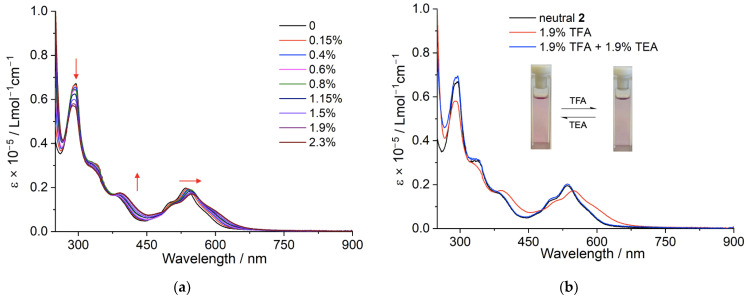
Variation of UV-Vis absorption spectra of **2** (1 × 10^−5^ M) in CH_2_Cl_2_ upon successive addition of TFA (**a**) at r. t. and reversible proton responsiveness (**b**).

As reported previously [9,16,24,45,46], azulene derivatives can undergo reversible protonation-deprotonation processes in the presence of trifluoroacetic acid (TFA) and triethylamine (TEA), respectively. To sort out the structural and electronic effects on stimuli-responsive and switching properties, the evolution of the UV-Vis absorption spectra of **1** (Figure 2) and **2** (Figure 3) in CH_2_Cl_2_ with increasing amounts of TFA was investigated. As depicted in Figure 2, the successive addition of TFA leads to a gradual reduction of the absorbances of electronic transitions peaked at 280 nm, 380 nm, and 577 nm with a concomitant bathochromic shift of the latter two absorption maxima towards 390 nm and 600 nm, respectively. This result is clearly evidenced by a fast and distinct color change from violet to light-green, pointing to newly formed azulenium species (Scheme 2) by protonation of two azulene rings of the triad **1** [9,16,46,47]. The formation of these two interconverting, optically different species is corroborated by the observed isosbestic points at 400 nm and 525 nm. No further noticeable spectral variation is observed upon protonation of **1** with a trace amount of TFA (1.9%, *v*/*v*). More strikingly, neutralization of the acidic solution of **1** with 1.9% TEA leads to a complete recovery of its original UV-Vis spectrum. Consequently, **1** displays a distinct reversible colorimetric pH response.

A similar spectral evolution is observed upon protonation of compound **2** but with much less pronounced changes than **1** (Figure 3). As the protonation process occurs in the five-membered ring of the azulene unit (Scheme 2), the annulation of azulene units to the PDI core via two five-membered rings undoubtedly affects proton responsiveness. The same holds true for the 1,3-substituted azulene-based polymers [9,48]. All these results suggest that non-substitution at the 1,3-positions of azulene is essential for an efficient protonation-deprotonation process. Nevertheless, the colorimetric reversibility is independent of the connectivity between azulene and PDI.

### 2.3. Electrochemical Properties

**Figure 4:**
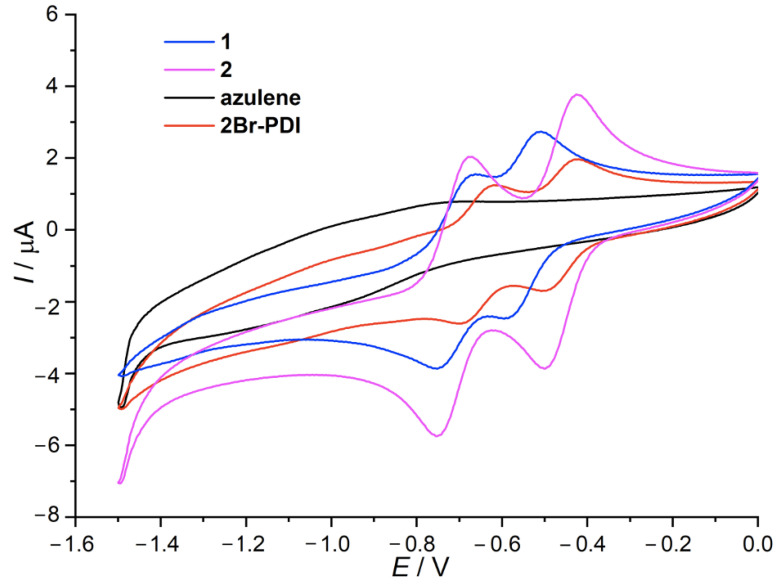
Cyclic voltammograms of **1** (blue), **2** (pink), azulene (black), and **2Br-PDI** (red) were measured in dichloromethane solution containing 0.1 M TBAPF_6_ as the supporting electrolyte at r.t., Pt working electrode and Ag/AgCl reference electrode at the scan rate of 100 mV s^−1^.

The electrochemical properties of **1** and **2** and their reference compounds were investigated by cyclic voltammetry at r.t. in CH_2_Cl_2_ (Table 1). Azulene itself shows an anodic peak at 1.19 V, while **2Br-PDI** undergoes two distinct reversible reductions at −0.47 V and −0.67 V, respectively (Figure 4). As expected, triad **1** displays one anodic peak (1.19 V) for the oxidation of the azulene core and two reversible processes at −0.55 V and −0.72 V due to the successive reductions of the PDI moiety. Apparently, the insertion of two azulene rings leads to no change in the oxidation potential but to negative shifts of the reduction potentials by 80 mV and 50 mV, respectively. The former is attributed to the negligible electronic interactions between the azulene rings and the PDI core due to the non-planar geometry (Figure S1). The latter can be accounted for by the electron-donating effect of azulene. In stark contrast, the fused counterpart **2** experiences one oxidation at 1.32 V and two reversible reductions at −0.46 V and −0.71 V, respectively. Compared to azulene (1.19 V) and PDI (−0.60 V and −0.79 V) [49], the annulation of these redox units in **2** gives rise to anodic shifts of both oxidation (by 130 mV) and reduction potentials (by 140 mV and 80 mV, respectively), indicating that both HOMO and LUMO are substantially stabilized by the extended π-conjugation between the resulting coronene core (the red part in Scheme 1) and two azulene rings. These results are further verified by TD-DFT calculations below. Remarkably, diazulenocoronene diimide **2** has a much lower HOMO-LUMO band gap (1.53 eV vs. 2.18 eV) and a lower-lying LUMO (−3.90 eV vs. −3.72 eV) with respect to its isomeric analogue dinaphthocoronene diimide [44]. As a result, **2** has a much higher-lying HOMO (−5.43 eV vs. −5.90 eV), indicating that the HOMO is substantially destabilized by 0.47 eV because of the electron-donating ability of azulene moieties. The reported non-fused azulene-based conjugates, however, show a HOMO destabilization of 0.16–0.2 eV [50] compared to their naphthalene counterparts. It can be deduced that in fused π-systems, the distinct electronic properties of azulene and naphthalene are expected to bring about a more effective influence on their optical properties and performance in organic electronics.

### 2.4. Computational Study

In order to characterize and verify the various electronic transitions and shed more light on the pH-response process and the intramolecular electronic interactions between the azulene and PDI units, DFT calculations were performed using the Gaussian 16 package at the B3LYP/6-31G(d,p) level of theory [51]. After geometry optimization, TD-DFT calculations were performed, solving 40 states. Absorption spectra were extracted using Gausssum 3.0 [52].

**Figure 5:**
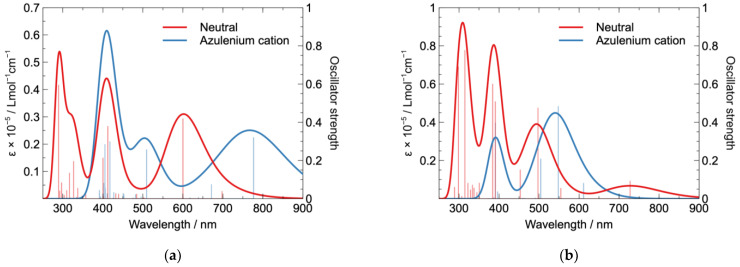
Computed absorption spectra and oscillator strengths of **1** (**a**) and **2** (**b**) before and after protonation.

The optimised molecular structure of **1** shows the non-planarity between two azulenes and the PDI core, whereas the newly formed diazulenocoronene core in the fused counterpart **2** is almost planar (Figure S1). The computed vertical absorption spectra of **1** and **2** (Figure 5) for the neutral and protonated states are in good agreement with the experimental results. The predicted energies and oscillator strengths (Tables S1–S4) at wavelength below 450 nm correspond to a manifold of π–π* transitions localized on PDI and azulene units, which compare well with their absorption spectra (Figure 1).

**Figure 6:**
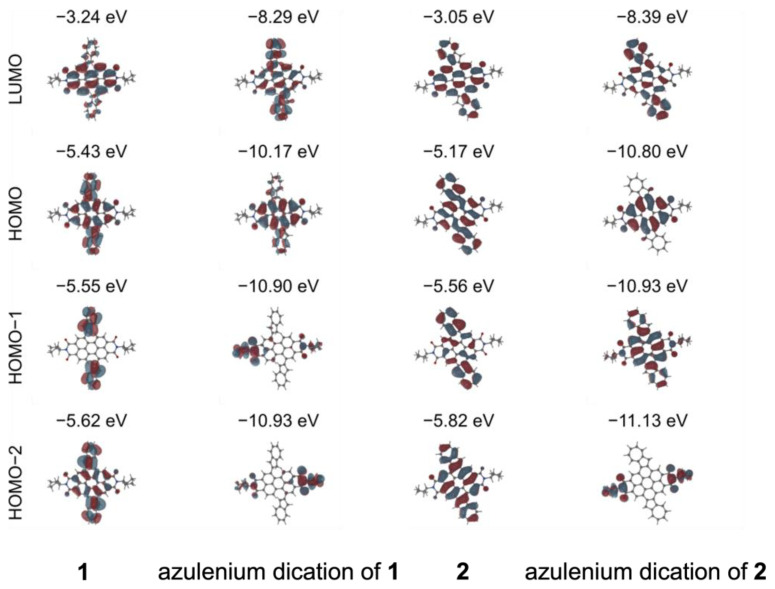
Frontier molecular orbitals of **1** (**left**) and **2** (**right**) and their azulenium dications are involved in the low-energy transition.

In the low energy region, a transition with low oscillator strength is found around 700 nm, which is attributed to the S_0_→S_1_ excitation dominated by one-electron transfer from HOMO→LUMO (76% for **1** and 93% for **2**, Tables S1 and S3). The next largest oscillator strength belongs to the transition at 600 nm for **1** and 500 nm for **2**, respectively. It is dominated by a one-electron HOMO-2→LUMO promotion (75% for **1** and 59% for **2**, Tables S1 and S3). As illustrated in Figure 6, for the HOMO and HOMO-2 of **1**, the electron density coefficients are largely localized on the azulene units with some extension to the adjacent PDI core; however, the LUMO is centered mostly on the PDI core. As a result, **1** exhibits a pronounced intramolecular charge-transfer (ICT) character as electron density is moved from the azulene units to the PDI core. In contrast, the MO coefficients for **2** are distributed over the whole diazulenocoronene backbone; therefore, the low-energy transitions have no directional ICT character. It is worth noting that the measured broad lowest-energy absorption band peaked at 577 nm for **1** and 535 nm for **2**, respectively, and is characterized by a combination of HOMO/HOMO-2→LUMO excitations. The calculated HOMO-LUMO gap amounts to 2.19 eV for **1** and 2.12 eV for **2** (Figure 6), which agrees well with the measured values (Table 1) based on their absorption spectra.

Upon protonation, the calculated vertical absorption spectra of **1** and **2** clearly display pronounced redshifts of the absorption bands (Table S3) due to the formation of azulenium cations. For the azulenium cation of **1**, the lowest-energy transition peaked at 776 nm is attributed to one-electron excitation (97%, Table S2) from the HOMO localized on the PDI core to the LUMO which is strongly extended to the azulene units, indicative of an ICT character leading to a HOMO-LUMO gap of 1.88 eV. On the contrary, for the azulenium cation of **2**, the longest wavelength absorption peaked at 611 nm shows only weak oscillator strength, whereas the strong 548 nm absorption is dominated by the one-electron excitation (92%, Table S4) from HOMO-1 delocalized on the dication diazulenocoronene core to the LUMO with a larger contribution of two cationic azulene units.

## 3. Materials and Methods

Air- and/or water-sensitive reactions were conducted under nitrogen and using dry, freshly distilled solvents. Chemicals used for the synthesis of the compounds were purchased from commercial suppliers (Sigma-Aldrich, Buchs, Switzerland, TCI, or Alfa Aesar). 1,7-Dibromo-*N*,*N*′-bis-(1-ethylpropyl)-perylene-3,4,9,10-tetracarboxylic diimide (**2Br-PDI**) [43] and 2-(azulen-2-yl)-4,4,5,5-tetramethyl-1,3,2-dioxaborolane [24] were prepared as described in the literature.

UV-Vis-NIR absorption spectra were recorded on a Perkin Elmer Lambda 900 UV/Vis/NIR spectrometer (Perkin Elmer, Schwerzenbach, Switzerland), and UV-vis absorption spectra were recorded on Varian Cary-100 Bio-UV/VIS (Agilent Technologies, Basel, Switzerland). ^1^H NMR spectra were recorded on a Bruker Avance 300 (300 MHz) spectrometer (Bruker, Fällanden, Switzerland). Chemical shifts are reported in parts per million (ppm) and are referenced to the residual solvent peak (CD_2_Cl_2_, *δ* ^1^H = 5.32 ppm). The following abbreviations were used: s (singlet), d (doublet), t (triplet), and m (multiplet). High-resolution mass spectra (HR-MS) were obtained on a Thermo Fisher LTQ Orbitrap XL using Nano Electrospray Ionization (Thermo Fisher Scientific, Basel, Switzerland).

Cyclic voltammetry (CV) was performed in a three-electrode cell equipped with a Pt working electrode, a glassy carbon counter-electrode, and an Ag/AgCl reference electrode. The electrochemical experiments were carried out under an oxygen-free atmosphere in dichloromethane with TBAPF_6_ (0.1 M) as a supporting electrolyte.

Synthesis of **1**: **2Br-PDI** (103 mg, 0.15 mmol), 2-(4,4,5,5-tetramethyl-1,3,2-dioxaborolan-2-yl)azulene (114 mg, 0.45 mmol), Cs_2_CO_3_ (293 mg, 0.9 mmol), and Pd(PPh_3_)_4_ (69 mg, 0.06 mmol) were added to a three-necked flask (100 mL) and flushed with N_2_. The toluene/H_2_O (20 mL/10 mL) was added into the flask and refluxed under N_2_ for 24 h. The reaction was quenched with aqueous 2M HCl (0.6 mL), extracted with toluene, washed with water and brine, and dried over Na_2_SO_4_. The obtained crude product was purified by column chromatography on silica gel eluting initially with CH_2_Cl_2_ to remove the unreacted azulene precursor and some by-products, and then with a mixture of heptane and ethyl acetate (gradient from 9:1 to 5:1(*v*/*v*)) to give the desired product with tiny impurities. An analytically pure product was obtained by the subsequent column chromatography on silica gel eluting with a mixture of CH_2_Cl_2_ and ethyl acetate (gradient from 1:0 to 9:1(*v*/*v*)) (25 mg, 21%). ^1^H NMR (300 MHz, CD_2_Cl_2_) *δ* 8.78 (s, 2H), 8.34 (d, *J* = 9.4 Hz, 4H), 7.97 (s, 4H), 7.64 (t, *J* = 10.1 Hz, 2H), 7.59 (s, 4H), 7.24 (t, *J* = 9.8 Hz, 4H), 5.06–4.95 (m, 2H), 2.31–2.14 (m, 4H), 1.96–1.81 (m, 4H), 0.89 (t, *J* = 7.4 Hz, 12H). HR–MS (ESI, positive): *m*/*z* calcd for [M + H]^+^ 783.3217, found 783.3207.

Synthesis of **2**: 2,3-dicyano-5,6-dichloro-1,4-benzoquinone (36 mg, 0.16 mmol) was added into a solution of **1** (31 mg, 0.04 mmol) in CH_2_Cl_2_ (20 mL) at r.t. and stirred for 3 h. The mixture was directly purified by column chromatography on neutral alumina using CH_2_Cl_2_ as eluant to afford an analytically pure product (12 mg, 40%). ^1^H NMR (300 MHz, CD_2_Cl_2_) *δ* 8.76 (s, 1H), 8.68 (s, 1H), 8.57 (s, 1H), 8.54 (s, 1H), 8.42 (t, *J* = 9.9 Hz, 2H), 8.13 (m, 2H), 7.95 (s, 1H), 7.89 (s, 1H), 7.77 (t, *J* = 9.3 Hz, 2H), 7.36 (t, *J* = 9.8 Hz, 2H), 7.31–7.21 (m, 2H), 5.04–4.93 (m, 2H), 2.27–2.11 (m, 4H), 1.91–1.78 (m, 4H), 0.86 (t, *J* = 7.4 Hz, 12H). HR-MS (ESI, positive): *m*/*z* calcd for [M + H]^+^ 779.2904, found 779.2888.

## 4. Conclusions

In summary, two azulene moieties were incorporated into a PDI core either through single bonds or by fusion, leading to the formation of **1** and **2**. The electronic structures of these target compounds were fully investigated by cyclic voltammetry and UV-Vis spectra and also verified by TD-DFT calculations. Both HOMO and LUMO energy levels can be tuned by the extent of conjugation between these redox-active units, giving rise to an electrochemical narrow HOMO-LUMO gap of ca. 1.5 eV. Additionally, they exhibit reversible colorimetric pH response, as corroborated by instant color switching between acidic and neutral environments. All these properties render them promising in the fields of organic electronics and sensing applications. Moreover, our findings present a new concept for manipulating the relative HOMO and LUMO energy positions of organic chromophores by fusing non-alternant PAHs.

## Figures and Tables

**Table 1:** Electrochemical data of the target compounds **1** and **2** together with reference compounds. Redox potentials (V) vs. Ag/AgCl in CH_2_Cl_2_.

Compounds	*E*^ox^ (V)	*E*_1/2_^red1^ (V)	*E*_1/2_^red2^ (V)	HOMO ^a^ (eV)	LUMO ^a^ (eV)	*E*_g_^CV^ (eV)	*E*_g_^opt^ (eV)
**azulene**	1.19			−5.31			1.74 ^c^
**2Br-PDI**		−0.47	−0.67		−3.90		2.27 ^c^
**1**	1.19	−0.55	−0.72	−5.36	−3.81	1.55 ^b^	1.90 ^c^
**2**	1.32	−0.46	−0.71	−5.43	−3.90	1.53 ^b^	2.00 ^c^

^a^ *E*_HOMO_ = −e (*E*_ox_ + 4.27), *E*_LUMO_ = −e (*E*_red_ + 4.27), *E*_ox_ = the onset oxidation potential, *E*_red_ = the onset reduction potential, Fc/Fc^+^ is 0.53 V relative to Ag/AgCl in CH_2_Cl_2_. ^b^ *E*_g_^CV^ = *E*_LUMO_ − *E*_HOMO_. ^c^ Optical band gap *E*_g_^opt^ is determined from the onset of the lowest energy electronic absorption in the corresponding UV-vis spectrum in solution.

## Data Availability

Not applicable.

## Supplementary Materials

The following supporting information can be downloaded at: https://www.mdpi.com/article/10.3390/molecules28186694/s1, Figure S1: The optimized structures of **1** (left) and **2** (right); Table S1: Energy, wavelength, oscillator strength (>0.01), and major molecular orbital contributions to transitions of **1** according to DFT calculations; Table S2: Energy, wavelength, oscillator strength (>0.01), and major molecular orbital contributions to transitions of the azulenium cation of **1** according to DFT calculations; Table S3: Energy, wavelength, oscillator strength (>0.01), and major molecular orbital contributions to transitions of **2** according to DFT calculations; Table S4: Energy, wavelength, oscillator strength (>0.01), and major molecular orbital contributions to transitions of the azulenium cation of **2** according to DFT calculations; Figure S2. The molecular orbitals involved in the main transitions of **1**, **2** and their azulenium dications; ^1^H NMR spectra of **1** and **2** in CD_2_Cl_2_.
