# Supplementary material for: Merging of Azulene and Perylene Diimide for Optical pH Sensors
Source: Molecules. 2023 Sep 19;28(18):6694. doi: 10.3390/molecules28186694 (PMC10537133; doi:10.3390/molecules28186694)
Supplement: Supplementary file 1 [file molecules-28-06694-s001.zip › molecules-2598937-supplementary.pdf]

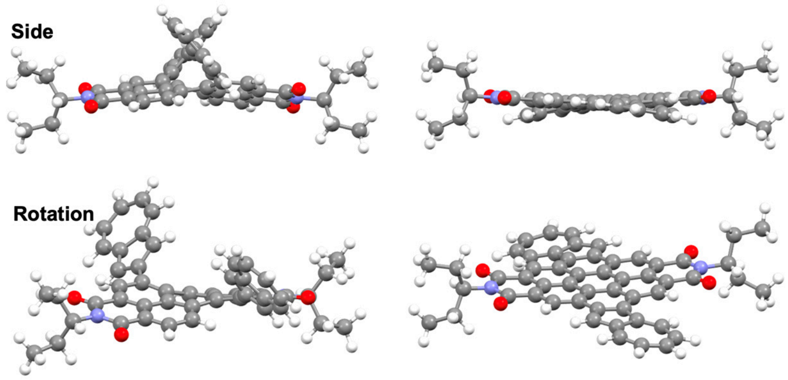

Figure S1 The optimized structures of **1** (left) and **2** (right).

Table S1. Energy, wavelength, oscillator strength (> 0.01), and major molecular orbital contributions to transitions of **1** according to DFT calculations.

State (S_n)	Energy (cm-1)	λ (nm)	Oscillator strength	Major MO contributions (%)
S_1	14330	697.8	0.0395	H-2→LUMO (22%), HOMO→LUMO (76%)
S_3	16662	600.2	0.4195	H-2→LUMO (75%), HOMO→LUMO (23%)
S_6	20641	484.5	0.0248	H-3→LUMO (87%)
S_7	22768	439.2	0.025	H-4→LUMO (45%), H-2→L+2 (13%) HOMO→L+2 (34%)
S_8	23131	432.3	0.028	H-2→L+1 (18%), H-1→L+2 (12%) HOMO→L+1 (62%)
S_9	24247	412.4	0.3794	H-4→LUMO (42%), H-1→L+1 (19%) HOMO→L+2 (37%)
S_{11}	24982	400.3	0.2142	H-2→L+2 (62%), H-1→L+1 (24%)
S_{20}	27994	357.2	0.0224	H-13→LUMO (18%), H-11→LUMO (73%)
S_{23}	28730	348.1	0.0149	H-3→L+1 (64%), HOMO→L+6 (12%)
S_{25}	29655	337.2	0.0562	H-13→LUMO (60%), H-11→LUMO (17%)
S_{30}	30593	326.9	0.1969	H-2→L+6 (13%), HOMO→L+6 (74%)
S_{33}	31607	316.4	0.1353	H-4→L+2 (62%), H-3→L+1 (14%) H-1→L+5 (14%)
S_{35}	32298	309.6	0.0445	H-2→L+6 (40%), H-1→L+5 (44%)
S_{37}	32961	303.4	0.0195	H-14→LUMO (22%), HOMO→L+7 (60%)
S_{38}	33687	296.9	0.085	H-2→L+6 (10%), H-1→L+7 (82%)
S_{39}	34233	292.1	0.0417	H-2→L+7 (74%), H-1→L+6 (11%)
S_{40}	34562	289.3	0.5956	H-4→L+2 (12%), H-3→L+4 (42%) HOMO→L+8 (19%)

Table S2. Energy, wavelength, oscillator strength (> 0.01), and major molecular orbital contributions to transitions of the azulenium cation of **1** according to DFT calculations.

State (S_n)	Energy (cm-1)	λ (nm)	Oscillator strength	Major MO contributions (%)
S_1	12877	776.6	0.3212	HOMO→LUMO (97%)
S_2	14894	671.4	0.0754	HOMO→L+1 (91%)
S_9	19631	509.4	0.2581	HOMO→L+4 (84%)
S_{10}	20184	495.4	0.0103	H-5→LUMO (89%)
S_{12}	20765	481.6	0.0228	H-1→L+1 (97%)
S_{15}	22095	452.6	0.0225	H-7→LUMO (36%), H-1→L+2 (56%)
S_{16}	22180	450.9	0.029	H-7→LUMO (45%), H-1→L+2 (42%)
S_{23}	23398	427.4	0.0345	H-9→LUMO (42%), H-8→LUMO (29%) H-6→L+1 (11%)
S_{26}	23943	417.7	0.2999	H-9→LUMO (37%), H-8→LUMO (45%)
S_{29}	24339	410.9	0.0271	H-12→LUMO (33%), H-10→LUMO (31%) H-1→L+4 (14%)
S_{30}	24620	406.2	0.0566	H-1→L+4 (71%)
S_{31}	24682	405.2	0.2853	H-7→L+1 (68%), H-2→L+4 (10%)
S_{32}	24864	402.2	0.0822	H-2→L+4 (70%)
S_{34}	24906	401.5	0.0173	H-5→L+2 (82%)
S_{36}	24973	400.4	0.0115	H-6→L+2 (32%), H-4→L+4 (41%)
S_{37}	25351	394.5	0.0113	H-12→LUMO (10%), H-11→LUMO (35%) H-10→LUMO (48%)
S_{39}	25532	391.7	0.0281	H-12→LUMO (12%), H-7→L+2 (56%)
S_{40}	25558	391.3	0.0451	H-12→LUMO (23%), H-11→LUMO (42%) H-7→L+2 (18%)

Table S3. Energy, wavelength, oscillator strength (> 0.01), and major molecular orbital contributions to transitions of **2** according to DFT calculations.

State (S_n)	Energy (cm-1)	λ (nm)	Oscillator strength	Major MO contributions (%)
S_1	13743	727.6	0.0933	HOMO→LUMO (93%)
S_3	18035	554.5	0.056	H-2→LUMO (28%), HOMO→L+2 (58%)
S_5	20105	497. 4	0.4756	H-2→LUMO (59%), H-1→L+1 (18%)<br>HOMO→L+2 (11%)
S_6	22075	453.0	0.1518	H-1→L+1 (65%), HOMO→L+2 (26%)
S_{11}	25595	390.7	0.509	HOMO→L+3 (81%)
S_{13}	26034	384.1	0.6012	H-3→LUMO (34%), H-2→L+2 (50%)
S_{19}	28512	350.7	0.0828	H-1→L+4 (64%), H-1→L+5 (26%)
S_{20}	29009	344.7	0.034	H-4→L+1 (15%), H-2→L+3 (13%), H-1→L+5 (49%)
S_{23}	29632	337.5	0.0571	H-11→LUMO (21%), H-3→L+2 (18%)<br>H-1→L+6 (34%)
S_{25}	29984	333.5	0.0731	H-3→L+2 (20%), H-2→L+3 (18%), H-1→L+6 (48%)
S_{28}	30410	328.8	0.047	H-11→LUMO (39%), H-8→LUMO (50%)
S_{31}	31025	322.3	0.0825	H-4→L+1 (13%), H-3→L+2 (32%), H-2→L+3 (44%)
S_{33}	31774	314.7	0.7775	H-4→L+1 (22%), H-1→L+5 (12%)<br>HOMO→L+7 (51%)
S_{35}	32587	306.9	0.0131	H-4→L+2 (48%), H-1→L+3 (10%)
S_{36}	33503	298.5	0.6914	H-14→LUMO (22%), H-4→L+1 (30%)<br>HOMO→L+7 (25%)
S_{40}	34632	288.8	0.0613	H-14→LUMO (17%), H-9→L+1 (12%)<br>H-7→L+1 (37%), H-3→L+3 (24%)

Table S4. Energy, wavelength, oscillator strength (> 0.01), and major molecular orbital contributions to transitions of the azulenium cation of **2** according to DFT calculations.

State (S_n)	Energy (cm-1)	λ (nm)	Oscillator strength	Major MO contributions (%)
S_1	16359	611.3	0.0821	HOMO→LUMO (95%)
S_2	18246	548.1	0.4839	H-1→LUMO (92%)
S_7	19831	504.3	0.2091	H-3→LUMO (88%)
S_{30}	25227	396.4	0.0375	H-1→L+4 (60%), HOMO→L+4 (25%)
S_{33}	25584	390.9	0.3977	H-1→L+4 (22%), HOMO→L+4 (57%)

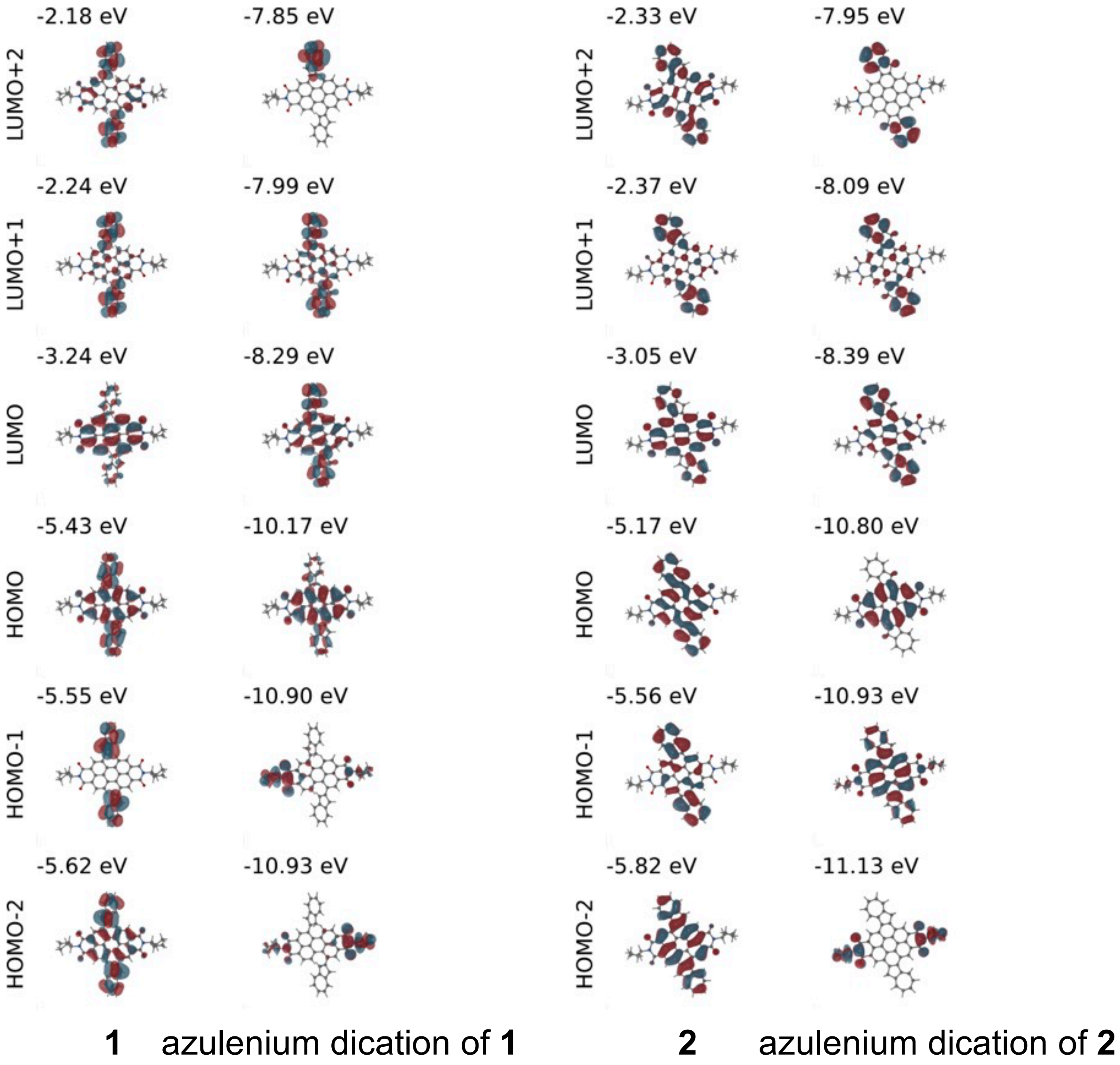

Figure S2. The molecular orbitals involved in the main transitions of **1**, **2** and their azulenium dications.

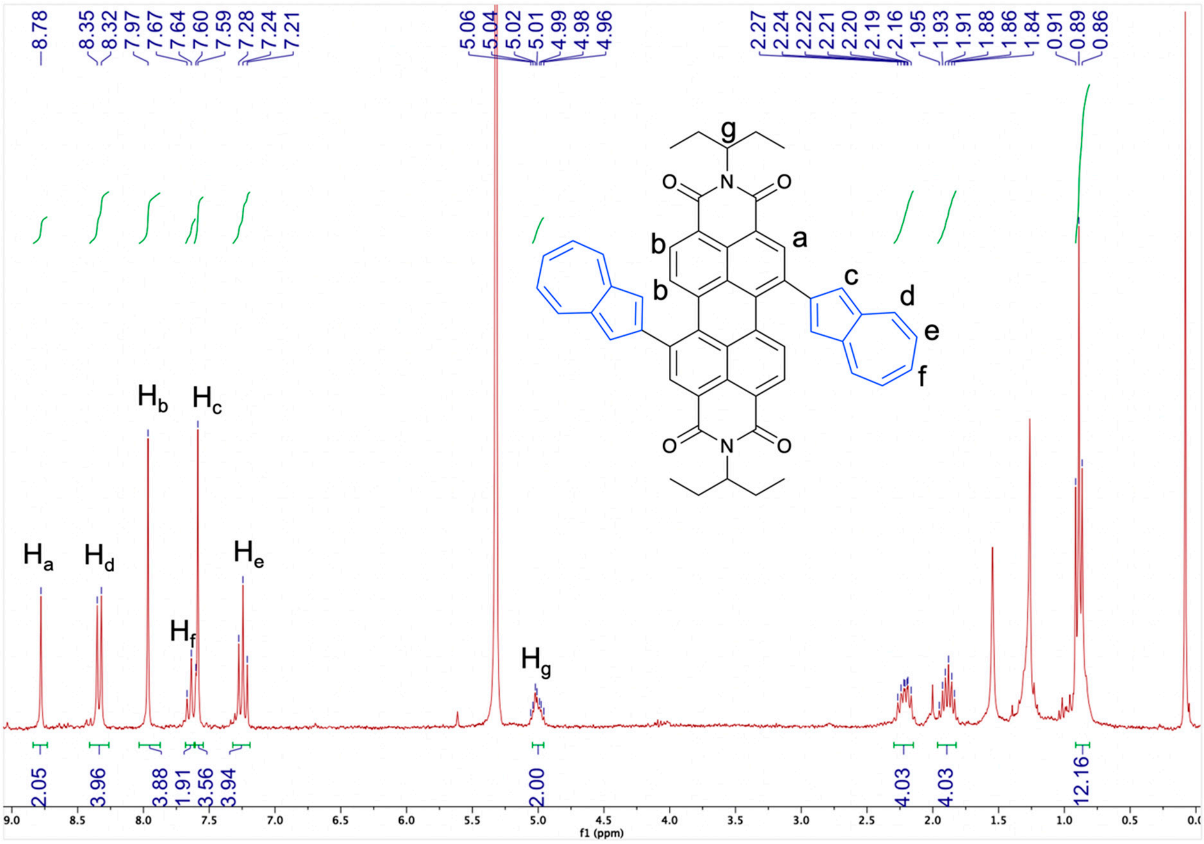

^{1}H NMR spectrum of compound **1** in CD_2Cl_2.

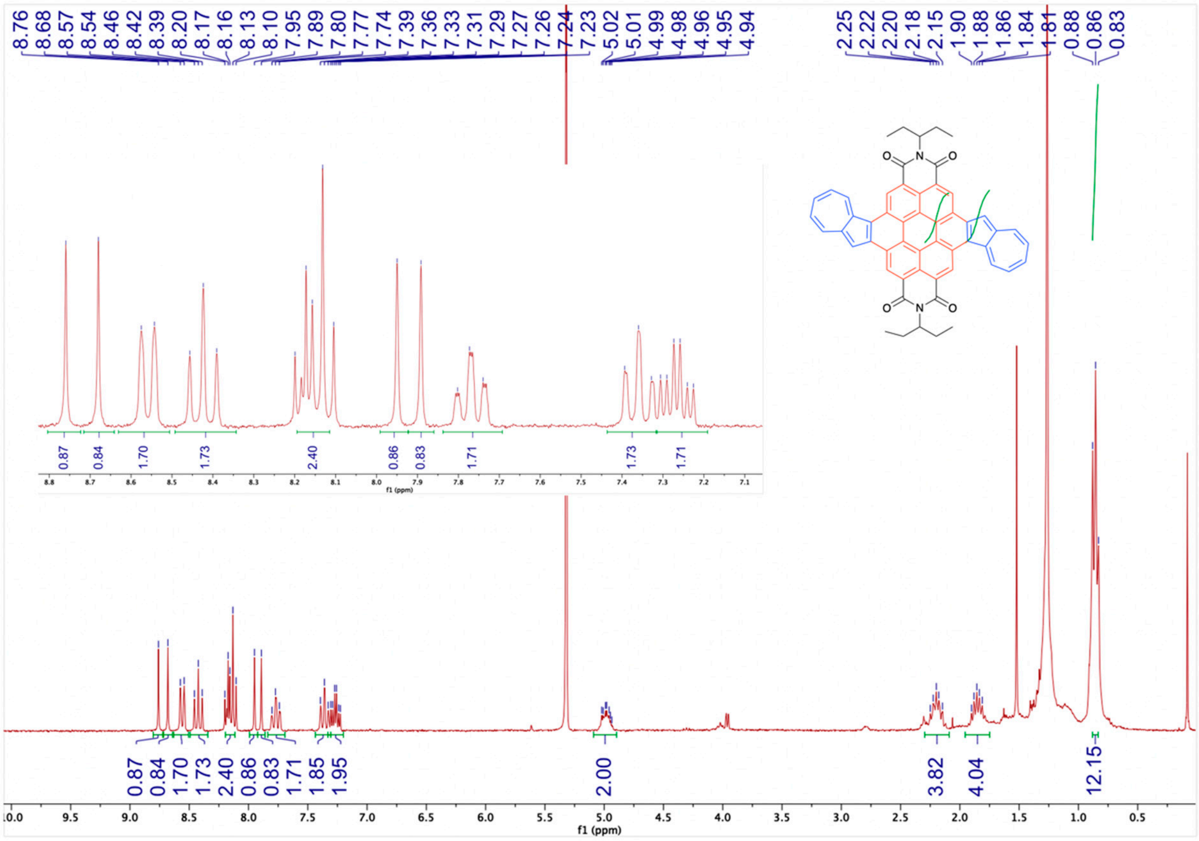

^{1}H NMR spectrum of compound **2** in CD_2Cl_2.
